# Analysis of the Ability of Different Allografts to Act as Carrier Grafts for Local Drug Delivery

**DOI:** 10.3390/jfb14060305

**Published:** 2023-06-01

**Authors:** Nicole Bormann, Aysha Schmock, Anja Hanke, Volker Eras, Norus Ahmed, Maya S. Kissner, Britt Wildemann, Jan C. Brune

**Affiliations:** 1 Julius Wolff Institut und BIH-Center für Regenerative Therapien und Center, Charité-Universitätsmedizin Berlin, Corporate Member of Freie Universität Berlin, Humboldt-Universität zu Berlin und Berlin Institute of Health, 13353 Berlin, Germany; nicole.bormann@bih-charite.de (N.B.); aysha.bonell@bih-charite.de (A.S.); maya.kissner@web.de (M.S.K.); britt.wildemann@med.uni-jena.de (B.W.); 2 German Institute for Cell and Tissue Replacement (DIZG, gemeinnützige GmbH), 12555 Berlin, Germany; a_hanke@dizg.de (A.H.); v_eras@dizg.de (V.E.); j_brune@dizg.de (J.C.B.); 3 Experimental Trauma Surgery, Department of Trauma, Hand and Reconstructive Surgery, Jena University Hospital, Friedrich Schiller University, 07747 Jena, Germany

**Keywords:** bone, infection, grafting material, allograft, antibiotics

## Abstract

Bone defects and infections pose significant challenges for treatment, requiring a comprehensive approach for prevention and treatment. Thus, this study sought to evaluate the efficacy of various bone allografts in the absorption and release of antibiotics. A specially designed high-absorbency, high-surface-area carrier graft composed of human demineralized cortical fibers and granulated cancellous bone (fibrous graft) was compared to different human bone allograft types. The groups tested here were three fibrous grafts with rehydration rates of 2.7, 4, and 8 mL/g (F(2.7), F(4), and F(8)); demineralized bone matrix (DBM); cortical granules; mineralized cancellous bone; and demineralized cancellous bone. The absorption capacity of the bone grafts was assessed after rehydration, the duration of absorption varied from 5 to 30 min, and the elution kinetics of gentamicin were determined over 21 days. Furthermore, antimicrobial activity was assessed using a zone of inhibition (ZOI) test with *S. aureus*. The fibrous grafts exhibited the greatest tissue matrix absorption capacity, while the mineralized cancellous bone revealed the lowest matrix-bound absorption capacity. For F(2.7) and F(4), a greater elution of gentamicin was observed from 4 h and continuously over the first 3 days when compared to the other grafts. Release kinetics were only marginally affected by the varied incubation times. The enhanced absorption capacity of the fibrous grafts resulted in a prolonged antibiotic release and activity. Therefore, fibrous grafts can serve as suitable carrier grafts, as they are able to retain fluids such as antibiotics at their intended destinations, are easy to handle, and allow for a prolonged antibiotic release. Application of these fibrous grafts can enable surgeons to provide longer courses of antibiotic administration for septic orthopedic indications, thus minimizing infections.

## 1. Introduction

In orthopedic and trauma surgery, infections have devastating consequences and are challenging to treat [1,2]. Surgical site infections (SSIs) are apparent worldwide, with reports that in England, deep wound infection after proximal femoral fracture incurred total costs of treatment per infected case of £24,410 compared with £7210 for patients without infection [3]. A recent study from the US reported a significant increase in healthcare costs over 16 months due to SSI after orthopedic surgeries, with a 64% increase in the two-year costs due to SSI [4]. Infections lead to longer hospital stays; in the case of SSI in hip fractures, the mean length of stay of patients was 76 days (50 days for superficial wound infections, 100 days for deep wound infections) [5]. Patients without infection had a mean length of stay of 22 days [5]. A longer length of stay and increased costs have also been reported for inpatient care in Germany [6], where the case costs of SSI groups were around €19,008 compared with €9040 for patients without infection. Overall, SSI can lead to increased mortality and morbidity, longer hospital stays, and increased economic burdens [6,7,8]. Thus, reducing infections in orthopedic surgery is important for both patients and clinicians. Treatment of a bone infection is difficult due to the administration of antibiotics systemically, which leads to low concentrations reaching the locally infected bone tissue [9]. The use of bone grafts has been suggested as a suitable carrier system for antibiotics, and these grafts can be loaded with different antimicrobial agents [10]. Access to autologous bone can be limited, which leads to more grafting sites and longer hospital stays [11]. Hence, allografts offer an alternative to autologous bone without the risk of donor site morbidity. In orthopedic surgery, the preference for allograft use is apparent, with allograft use increasing by 74.1% and autologous bone tissue use decreasing by 14.3% between 2008 and 2018 [12]. Depending on the surgical indications, allografts provide surgeons with a vast range of grafting materials that are available in various forms. These grafting materials are continuously being modified and improved to ensure successful transplantation. A common grafting material is demineralized bone matrix (DBM) derived from human bone. The material has been used in various surgical indications [13,14,15,16]. Allografts can also be mixed with other substances such as antibiotics to help treat patient infections [17,18,19]. The addition of such substances allows grafts to be used for prophylaxis and/or treatment by directly delivering antibiotics to the target site. This avoids high systemic levels of antibiotics and the putative generation of resistance or side effects. 

Delivery of antibiotics and liquids to a desired site of action can be difficult, as the fluids can be washed away during surgery and are often removed via natural processes. Commercially available cements loaded with antibiotics are frequently used in Europe to treat and prevent infections during surgery. These cements allow the local delivery of antibiotics to the surgical site [20]. However, cements are not resorbable and require a secondary surgery for removal [21]. Therefore, it is essential to develop antibiotic-impregnated bone grafts that are able to deliver and release antibiotics while being resorbable. The elution kinetics of DBM allografts with different antibiotics were previously analyzed [22]. In vitro testing of DBM putty displayed clinically relevant release kinetics, antimicrobial potency, and no cytotoxicity [22]. The use of allografts loaded with antibiotics was reported by Ketonis et al. [23], where vancomycin was tethered to cortical and cancellous bone chips and investigated regarding the adherence of bacteria after the bound graft was stored in PBS for 45 days [23]. Witsø et al. investigated the release of antibiotics from an antibiotic-impregnated cortical bone allograft over different periods of time [24]. The authors reported that in the in vitro study, the incubation time of graft and antibiotic influenced the amount of antibiotic released. Additionally, a rat in vivo study demonstrated that netilmicin, vancomycin, and rifampicin effectively eradicated perioperative contamination with *S. aureus* [24]. Due to the nature of allografts, many forms of bone allografts can be used in orthopedic surgery. Here, a high-absorption carrier graft was explicitly developed for drug loading and release. This newly developed graft was then investigated for its elution kinetics and compared with other standard bone allografts such as DBM granules, cortical granules, mineralized cancellous bone, and demineralized cancellous bone. The tested allografts were sterilized using a validated peracetic-acid-based process [25]. The absorption capacities and gentamicin release kinetics of the different human bone allografts were assessed.

## 2. Material and Methods

### 2.1. Allografts

The human allograft tissues used in this study were provided by the German Institute for Cell and Tissue Replacement (DIZG, gemeinnützige GmbH, Berlin, Germany). All human tissues were acquired from nonprofit tissue recovery partners after informed consent. Grafts were sterilized using a validated, GMP-conformable process and were approved as medicinal products under §21 and §21a of the German Medicinal Products Act. For sterilization, tissues were fully submerged in a validated tissue-preserving sterilization solution (2% peracetic acid, 96% ethanol, water for injection; ratio v/v/v 2/1/1) and incubated with constant agitation at low pressure and room temperature for 4 h [25]. Subsequently, tissues were rinsed in a washing process using water for injection.

### 2.2. Graft Absorption Capacity Testing

The allografts included in the absorption capacity testing (Supplementary Table S1) were the novel fibrous graft (Fiberfill^®^, DIZG, Berlin, Germany) consisting of demineralized cortical fibers and granulated cancellous bone at a rehydration rate of 2.7, 4, or 8 mL/g; DBM (1–2 mm); cortical granules (1–2 mm); low-, medium-, and high-density mineralized cancellous bone (weight per cubic centimeter volume (g/cc)); and demineralized cancellous bone (Spongioflex^®^, DIZG Germany). All grafts were weighed to a dry volume of 0.4 cc using a digital scale (KERN, Albstadt, Germany). Grafts were rehydrated with phosphate-buffered saline (PBS) to a final graft volume of 0.4 cc (Supplementary Table S1). A sieve was 3D printed for the absorption capacity testing (designed using onshape^®^, Boston, USA, and 3D printed by the DIZG using Form 2, Formlabs, Berlin, Germany). Rehydrated samples were centrifuged in the 3D-printed sieve (inner diameter 9 mm, height 6.3 mm) at 1000× *g* for 3 min to gravimetrically determine the absorptive capacities of the matrix and interstitial spaces. Total PBS absorption was calculated for all grafts and normalized to the original graft volume. Different sieves were used to control the graft geometry. Matrix-associated fluid was defined as the fluid that resisted 1000× *g* centrifugation for 3 min. Extracted fluid was considered to be of interstitial origin.

### 2.3. Elution Kinetics

**Figure 1:**
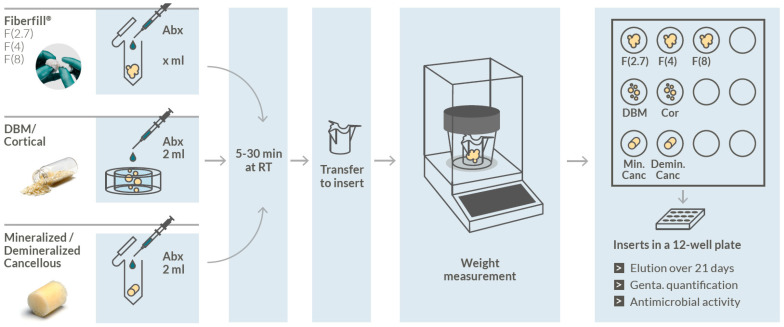
Schematic of the gentamicin elution experimental setup. Abbreviation: Abx, antibiotics.

For the graft elution parameters, the novel fibrous graft (Fiberfill^®^, DIZG Germany) with a rehydration rate of 2.7, 4, or 8 mL/g (F(2.7), F(4), F(8)); DBM granules (DBM) (1–2 mm); cortical granules (Cor) (1–2 mm); mineralized cancellous bone (Min. Canc); and demineralized cancellous bone (Demin. Canc; Spongioflex^®^, DIZG Germany) were tested. Depending on the allograft, different methods were used to mix the graft with 40 mg/mL gentamicin (Ratiopharm, Ulm, Germany) (Figure 1, Supplementary Table S2).

### 2.4. Fibrous Graft (Fiberfill^®^)

The fibrous grafts were weighed in 2 mL tubes, and gentamicin was added so that a final wet volume of 0.4 cc was achieved in every case. The grafts were mixed with a spatula to a homogeneous mass with no liquid remaining. This confirmed complete gentamicin uptake. After incubation, the mixture was transferred into cell culture inserts (Ø 10 mm, 8 μm pore size, polycarbonate membrane, Nunc™, Thermo Fischer Scientific, Hennigsdorf, Germany) with a spatula.

### 2.5. DBM and Cortical Granules

The dry weights of DBM and cortical granules were determined using an OrganCulture Dish (BD Falcon, Heidelberg, Germany), and 2 mL gentamicin was added to each sample. After incubation, the grafts were transferred into cell culture inserts and, at the same time, the unabsorbed gentamicin was removed with a syringe. Each graft–gentamicin mixture resulted in a final wet volume of 0.4 cc.

### 2.6. Cancellous Bone and Demineralized Cancellous Bone (Spongioflex^®^)

The grafts (0.4 cc) were transferred to 5 mL tubes, dry weights were determined, and 2 mL of gentamicin was added to each sample. Samples were carefully transferred into cell culture inserts without compressing the grafts (preventing a loss of interstitial fluid).

### 2.7. Exposure Times and Release

All grafts (three human donors for each group based on informed consent for research use) were incubated for 30 min at room temperature with gentamicin solution. Additionally, F(2.7) and (8) and Min. Canc (each *n* = 3) were incubated for 5, 10, and 20 min to investigate time-dependent gentamicin uptake and elution. After incubation, grafts were transferred to cell culture inserts. To determine the amount of gentamicin absorbed, the graft weight was determined. Subsequently, the inserts were placed in a carrier plate (designed using onshape®, Boston, MA, USA, and 3D printed by the DIZG using Form 2, Formlabs, Berlin, Germany) and transferred to a 12-well plate (BD Falcon, Heidelberg, Germany) with 4.5 mL PBS (Gibco, Thermo Fischer, Hennigsdorf, Germany) preloaded and another 500 μL of PBS pipetted into each insert. Incubation was performed in an incubator at 37 °C, 5% CO_2_, and 95% humidity for 21 days. At time points 1 h, 4 h, 1 d, 2 d, 3 d, 4 d, 7 d, 14 d, and 21 d, the cell culture inserts were transferred to a new 12-well plate and stored at room temperature for 10 min to allow excess fluid to drain. The PBS solution was removed entirely from the wells, weighed, aliquoted, and stored at −20 °C. Fresh PBS was added, and samples were further incubated. After 21 days of elution, the inserts with the grafts were placed in a 24-well plate and stored at 4 °C until the zone of inhibition test.

### 2.8. Gentamicin Quantification

Quantitative gentamicin analysis was conducted by the Berlin-Charité Vivantes GmbH laboratory where 500 μL measures of the samples were used. The analysis was performed using the GENT2 Roche/Hitachi cobas^®^c system (Roche GmbH, Mannheim, Germany), based on the kinetic interaction of microparticles in a solution (KIMS). The functional sensitivity of this assay is 0.4 μg/mL (0.84 μmol/L). In this assay, gentamicin antibody is covalently coupled to microparticles and the drug derivative is linked to a macromolecule. The kinetic interactions of the microparticles in the solution can be induced by the binding process of the drug conjugate to the antibody on the microparticles. This is inhibited due to the presence of gentamicin. The drug conjugate and the gentamicin in the serum sample compete for the binding sites of the gentamicin antibody on the microparticles. Thus, the resulting kinetic interaction of the microparticles is indirectly proportional to the amount of gentamicin present in the sample.

### 2.9. Zone of Inhibition Test (ZOI)

*Staphylococcus aureus* solution (ATCC 25923, McFarland 1) was prepared, diluted 1:2 with PBS (5 × 10^6^ CFU/mL), and plated with cotton swabs on Columbia agar with sheep blood (Oxoid, Wesel, Germany). Test filters (Ø 6 mm, area: 0.28 cm^2^, Oxoid, Germany) were placed on the plates and 15 μL was pipetted from each elution sample (*n* = 3 per group). In addition, after 21 days of elution, each graft was placed on an agar plate. The plates were incubated overnight at 37 °C, photographed, and the inhibition zones were measured using ImageJ software (1.41.o, National Institute of Health, Bethesda, MD, USA).

### 2.10. Statistics

Data are displayed as mean ± SD. Statistical analysis was performed using GraphPad Prism software version 8.2.1 (San Diego, CA, USA). The significance level (alpha set to 0.05) was determined using the ordinary one-way ANOVA with Tukey’s multiple comparisons test or a two-way ANOVA with Tukey’s multiple comparisons test.

## 3. Results

### 3.1. Liquid Absorption Capacity

**Figure 2:**
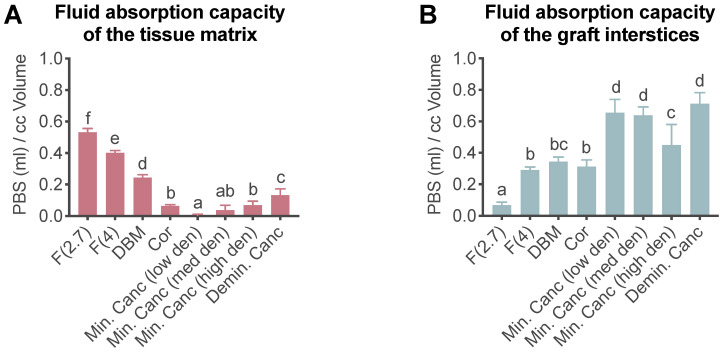
Compact letter display (CLD) graphs portraying pairwise differences between allografts for (**A**) total fluid absorbed into the matrix and (**B**) interstitial spaces. Data represented as mean ± SD *n* = 4–8: 8 for low-density cancellous bone, 4 for high-density cancellous bone and demineralized cancellous bone, and 6 for the other groups. Statistical analysis (Supplementary Table S3) was performed using the ordinary one-way ANOVA with Tukey’s multiple comparisons test (*p* ≤ 0.05). Significances are displayed using the compact letter display. Groups that do not share the same letter differed significantly from each other. F(2.7) and F(4): fibrous allograft with rehydration ratio of 2.7/4 mL/g; DBM: demineralized bone matrix granules; Cor: cortical granules; Min. Canc: mineralized cancellous bone (low-, med-, high-den = density); Demin. Canc: demineralized cancellous bone.

The PBS absorption capacity assay was used to investigate whether the grafts were able to absorb fluid into either the extracellular matrix or the graft spaces. The release kinetics of fluids may be affected by their absorption into the extracellular matrix or the graft spaces. Here, F(2.7) and F(4) demonstrated significantly superior binding capacity of PBS within the matrix compared to all other tested grafts (Figure 2, Supplementary Table S3). However, when looking at the absorption capacities of the graft interstices, cancellous bone displayed the highest absorption of PBS.

### 3.2. Graft Elution and Antimicrobial Activity

#### 3.2.1. Rehydration Time: 30 min

Due to the different uptake capacities of the grafts, the loaded amounts of gentamicin varied and values were calculated from the respective determined weights of the grafts before and after loading.

**Figure 3:**
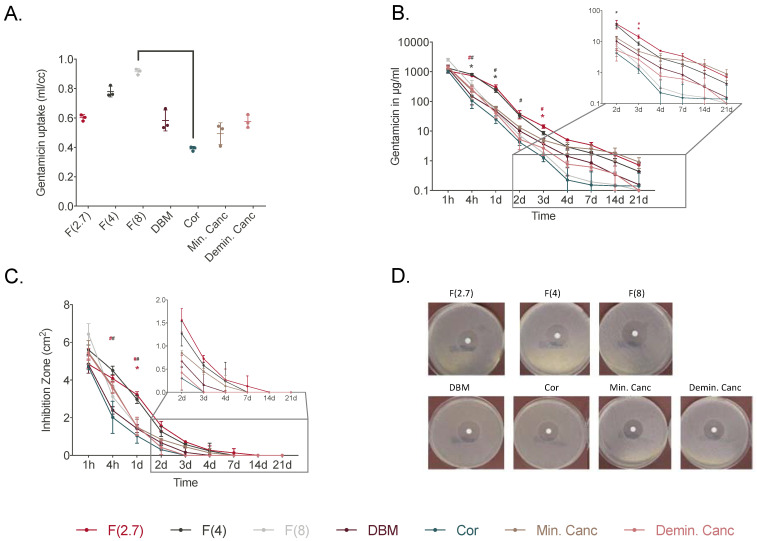
(**A**) Gentamicin uptake in different allografts calculated via the mass differences. (**B**) Quantitative determination of gentamicin after elution from different grafts in μg/mL (*n* = 3). (**C**) Inhibition zones of the respective grafts (*n* = 3). (**D**) Images of the ZOI tests of the respective grafts at 1 h. Significant differences of F(2.7) and F(4) to Min. Canc are shown by * and to DBM by #. The statistical significances for the other groups are presented in Supplementary Tables S4 and S5. Data are represented as mean ± SD. Statistical analysis was performed using the two-way ANOVA with Tukey’s multiple comparisons test (*p* ≤ 0.05).

The F(4) and F(8) groups showed the highest gentamicin uptake compared to all other grafts (Figure 3A). The elution of gentamicin varied among the different grafts when they were rehydrated for 30 min. The F(2.7) and F(4) groups displayed higher elution from 4 h and continuously over the first 3 days (Figure 3B, Supplementary Table S4). At day 3, F(2.7) displayed significantly higher gentamicin elution compared to the other allografts except for F(4). The ZOI testing of the individual eluates showed that F(2.7) and F(4) had significantly larger inhibition zones at 4 h compared to DBM. At day 1, F(2.7) displayed significantly larger inhibition zones compared to all grafts except for F(4) and demineralized cancellous bone (Figure 3C,D, Supplementary Table S5). Overall, when grafts were incubated with gentamicin for 30 min, groups F(2.7) and F(4) showed high gentamicin elution values leading to larger ZOI.

#### 3.2.2. Varying Rehydration Times: 5 to 30 min

**Figure 4:**
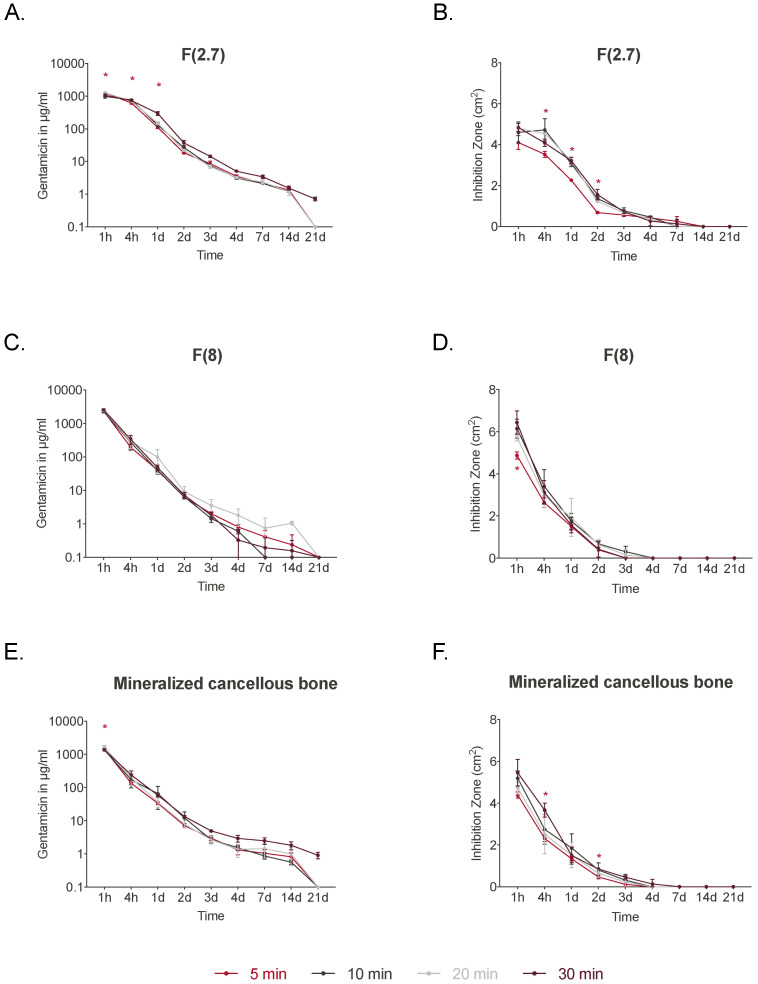
Gentamicin elution from the respective grafts with different incubation times and ZOI test results. (**A**) Elution of F(2.7) and (**B**) ZOI test results for F(2.7) with different incubation times. (**C**) Elution of F(8) and (**D**) ZOI test results for F(8) with different incubation times. (**E**) Elution of mineralized cancellous bone and (**F**) ZOI test results for mineralized cancellous bone with different incubation times (*n* = 3). Significant differences for the 5 min incubation time group compared to the 30 min group are shown with *. Data are represented as mean ± SD. Statistical analysis was performed using the two-way ANOVA with Tukey’s multiple comparisons test (*p* ≤ 0.05).

A graft’s absorbance capabilities may influence its ability to elute clinically relevant concentrations of antibiotics. Thus, different gentamicin incubation times were tested with the different bone grafts (Figure 4). Incubation times of 5, 10, 20, and 30 min were tested for F(2.7), F(8), and Min. Canc. Generally, incubation time had a minor impact on graft elution. Elution of gentamicin from F(2.7) at 1 h was significantly lower for the 5 min incubation compared to the 10 min and 30 min incubations, and was significantly lower than for the 30 min incubation after 1 day (Figure 4A). From day 2, no significant differences were observed for any of the groups. This was confirmed in the ZOI testing, in which eluates of F(2.7) incubated for 5 min resulted in smaller ZOI over 2 days (Figure 4B and Supplementary Table S6) compared to the 10 and 20 min treatments. No differences were observed at later time points. F(8) displayed no significant differences in gentamicin elution with different incubation times (Figure 4C). However, ZOI testing displayed a significantly smaller inhibition zone at 1 h after 5 min of incubation when compared to 20 min of incubation (Figure 4D). No differences were observed for the other incubation times after 1 h. Min. Canc showed a significantly lower elution of gentamicin at 1 h when grafts were incubated for 5 min compared to 20 min (Figure 4E). Smaller inhibition zones were observed at 4 h and 2 days for the 5 min incubation treatment group compared to the 30 min incubation group (Figure 4F). Over 21 days, the gentamicin exposure time did not influence the elution kinetics of F(2.7), F(8), or Min. Canc. After 21 days of elution, the grafts were placed directly onto agar plates plated with *S. aureus* (Supplementary Figure S1) and some grafts still displayed inhibition zones around the grafts, especially Min. Canc and Demin. Canc.

## 4. Discussion

During surgical treatment, bacteria can colonize bone and orthopedic implants, leading to infection. This requires surgeons to consider suitable grafting materials that can be used to treat bone defects but also contain antibiotics for prophylaxis or for treating patients suffering from infections. The choice of bone grafting material, in addition to effective antibiotic prophylaxis, is important and depends on the location, size, and type of bone defect. Generally, commercially available bone replacement materials that include antibiotics are based on bone cements and collagen [26]. High concentrations of antibiotics are required at the infection site, and a good local drug-release system is needed to achieve this [27]. The techniques used for delivering drugs to patients have evolved over the years. This has led to methods like the application of microneedles and clay-based composites which are able to deliver a sustained release of drugs through the skin [28]. Polymethylmethacrylate (PMMA) has also been used and mixed with antibiotics as a local drug-release system [29]. However, PMMA is not resorbable and requires a secondary surgery for removal [21]. Using resorbable grafts loaded with antibiotics prevents the need for a second surgery. If antibiotic-loaded grafts are capable of promoting bone healing while preventing infections, it will provide patients with an alternative option. Therefore, developing improved grafts that can absorb and retain antibiotics will be advantageous to both surgeons and patients. This study examined the loading and release of an antibiotic from different human allografts used for bone regeneration and a specially designed high-absorbency, high-surface-area carrier graft composed of demineralized cortical fibers and granulated cancellous bone. When compared to the other bony allografts, the new fibrous graft revealed the highest liquid absorption with a prolonged gentamicin release. A high antimicrobial activity was found for the eluate samples, with the highest activity observed for the 1 h samples and a prolonged activity notes for the fibrous grafts.

As previously shown, bone allografts have good antibiotic-uptake capabilities. Winkler et al. [27] reported that purified bone may store up to 10 times more vancomycin than cement. Reports have shown that bone grafts can store large amounts of antibiotics [30,31,32]. This emphasizes the significance of developing grafts that have the capacity to absorb and elute antibiotics for patient treatment. For the first time, this study evaluated the ability of different types of bone allograft to absorb and elute gentamicin for up to 21 days. This study sought to provide surgeons with a better understanding of the type and formulation of bone grafts that can be used to treat bone defects and prevent infection. The bone allografts used here differed in their particle size, shape, and processing procedure.

During demineralization, the bone is decalcified while preserving collagen and noncollagenous proteins [33]. Collagen polypeptides have been reported to have good moisture absorption and retention capabilities [34]. A study of a collagen/demineralized bone powder scaffold soaked in phosphate-buffered saline for 24 h reported particle swelling [35]. Preliminary experiments showed that the demineralization procedure can lead to swelling of the DBM particles (Supplementary Figure S2). This can be explained by the fact that the graft’s protein matrix is exposed during the demineralization process. According to the preliminary experiments, the rehydration of DBM causes fluid to accumulate between the collagen fibers, leading to an average 36% increase in the volume of the whole particle. This was calculated using the assumption of a spherical structure. This emphasizes the importance of the processing procedure and the potential effects it has on the grafts’ ability to absorb higher volumes of liquid. This phenomenon may not occur in mineralized samples. The higher PBS and gentamicin uptake by demineralized bone grafts such as the fibrous graft, DBM, and demineralized cancellous bone demonstrates this. Particles in a fiber form are more suitable for the adhesion of liquids due to the high surface-to-volume ratio of particles. This led to the development of the high-absorption carrier graft (Fiberfill^®^). This novel graft demonstrated the ability to absorb into the matrix and elute larger volumes of liquid compared to other bone allografts. Higher absorption into the tissue matrix may be responsible for prolonged elution, as absorption of fluids into the graft interstices may lead to a faster release when compared to absorption into the extracellular matrix. The novel fibrous graft and demineralized cancellous bone displayed good antibiotic absorption and elution properties with active gentamicin in the zone of inhibition test with *S. aureus*. Incubating the allografts with antibiotics for 10, 20, or 30 min did not seem to affect the elution properties, although incubating for 5 min resulted in significantly lower elution within the first day. Previous reports have shown that the length of time used for incubation of grafts and antibiotics influences the amount of antibiotic released [24]. In our tests, longer incubation times did not result in large differences in regard to the gentamicin elution over a 21-day period. It is important to note that this study only tested perioperatively practical incubation times of 5, 10, 20, and 30 min. The time points tested here might be suitable for incubation during surgery, while the incubation time points of 1, 10, and 100 h used in a previous study [24] might not be suitable for perioperative application. The low impact of the incubation time on elution means that the fibrous graft will be sufficiently loaded with antibiotics within 5 to 10 min. This also provides safety because there are no time restrictions that surgeons need to adhere to, as high local gentamicin concentrations will be reached after 5 min of incubation. Additionally, the novel fibrous graft is easy to handle, was tested with clinically used gentamicin solution, and has previously shown no systemic toxicity [36].

The elution kinetics presented in this study were in line with previous reports. During the initial 72 h, the tested fibrous grafts released a large proportion of the loaded gentamicin. This phenomenon is typically referred to as “burst release” [37,38] and leads to higher initial drug delivery, helping to tackle the initial bacterial contamination. The minimum inhibitory concentration (MIC) of gentamicin against *S. aureus* has been reported to be between 0.125 and 0.25 μg/mL [39]. The F(2.7) group tested in our study continued to elute gentamicin at concentrations higher than the reported MIC for *S. aureus* for up to 21 days in an experimental setting. The novel fibrous graft’s burst release and long-term elution characteristics may contribute to the attainment of a high local concentration to target the MICs of microorganisms.

The antibacterial activity observed was similar to that seen in other studies. In previous studies, PMMA loaded with vancomycin displayed bioactivity for 48 h against *S. aureus* [40]. A 48 h effectiveness against *S. aureus* was also described for a glass polyalkenoate cement loaded with vancomycin [41]. Antibiotic-loaded allografts may provide a suitable option for treating infections, and a clinical study using antibiotic-loaded allografts in previously infected patients reported that 46/48 patients remained free from infection for 1–7 years postsurgery [42]. An interest in antibiotic-loaded allografts was also reported in a study that used tethered-vancomycin allografts from which no release could occur into the surroundings. The grafts were then stored in PBS for 45 days, and the antibiotic-loaded grafts still significantly decreased the adherence of bacteria [23]. Taken together, depending on the treatment goal, infection eradication or prophylaxis, the amount of antibiotic loaded into the allograft matrix can be adapted by the surgeon. Based on the individual situation, higher amounts might be used to successfully eradicate infection with the risk of impaired bone formation, as seen in an animal study [43]. Nevertheless, in the case of prophylactic use, less antibiotic is required, and the concentration should not affect bone regeneration.

The study’s limitations are the in vitro situation: fluids in vivo may react differently and fluid may leave the intended target site. This limitation was reduced by using a full PBS exchange in the elution experiment at the tested time points to mimic a high fluid exchange. A full exchange of the elution medium has been shown to lead to faster elution of gentamicin compared to a 50% medium change, which led to an earlier loss of inhibitory capacity [22].

## 5. Conclusions

To conclude, the novel fibrous bone allograft can be easily mixed with clinically used gentamicin solution and displays good absorption kinetics. The fibrous graft does not need a special gentamicin solution or require specific mixing techniques. This provides surgeons with a grafting material that can be used for prophylaxis or to treat patients suffering from infections. However, further studies are required to test such allografts with different antibiotics.

## Data Availability

Data are available on request.

## Supplementary Materials

The following supporting information can be downloaded at: https://www.mdpi.com/article/10.3390/jfb14060305/s1, Figure S1: Zone of inhibition (ZOI) test after 30 min of incubation: Gentamicin elution from the respective grafts after 21 days of elution; Figure S2: Microscopy images of DBM particles before (A,C, left) and after (B,D, right) rehydration with water (6.3× magnification, scale bar = 200 μm); Table S1: Grafts used for the absorption capacity testing with PBS; Table S2: Grafts used for the gentamicin elution testing; Table S3: Statistical significance of total fluid absorbed in the matrix (lower part in red) and interstitial spaces (upper part in blue). The exact *p*-values are given. Fibrous grafts absorbed significantly more fluid than any other group; Table S4. Statistical significance of gentamicin elution by different bony grafts over a 21-day period. The exact *p*-values are given. No significant differences were observed after day 7. ns: not significant (*p* > 0.05); F(2.7): Fibrous graft 2.7 mL/g, F(4): Fibrous graft 4 mL/g; F(8): Fibrous graft 8 mL/g; Cor: cortical granules; Min. Canc: cancellous bone; DBM: demineralized bone matrix; Demin. Canc: demineralized cancellous bone; Table S5: Statistical significance of ZOI testing with different bony grafts over a 21-day period. The exact *p*-values are given. No significant differences were observed after day 3. ns: not significant (*p* > 0.05); F(2.7): Fibrous graft 2.7 mL/g, F(4): Fibrous graft 4 mL/g; F(8): Fibrous graft 8 mL/g; Cor: cortical granules; Min. Canc: cancellous bone; DBM: demineralized bone matrix; Demin. Canc: demineralized cancellous bone; Table S6: Statistical significance of gentamicin elution of Fibrous graft 2.7 mL/g with different incubation times over a 21-day period. The exact *p*-values are given. No significant differences were observed after day 1. ns: not significant (*p* > 0.05).

## References

[1] Moriarty T.F., Metsemakers W.J., Morgenstern M., Hofstee M.I., Vallejo Diaz A., Cassat J.E., Wildemann B., Depypere M., Schwarz E.M., Richards R.G. (2022). Fracture-related infection. Nat. Rev. Dis. Prim..

[2] Kennedy D.G., O’Mahony A.M., Culligan E.P., O’Driscoll C.M., Ryan K.B. (2022). Strategies to Mitigate and Treat Orthopaedic Device-Associated Infections. Antibiotics.

[3] Pollard T.C., Newman J.E., Barlow N.J., Price J.D., Willett K.M. (2006). Deep wound infection after proximal femoral fracture: Consequences and costs. J. Hosp. Infect..

[4] Shapiro L.M., Graham L.A., Hawn M.T., Kamal R.N. (2022). Quality Reporting Windows May Not Capture the Effects of Surgical Site Infections After Orthopaedic Surgery. J. Bone Jt. Surg. Am. Vol..

[5] Edwards C., Counsell A., Boulton C., Moran C.G. (2008). Early infection after hip fracture surgery: Risk factors, costs and outcome. J. Bone Jt. Surg. Br..

[6] Eckmann C., Kramer A., Assadian O., Flessa S., Huebner C., Michnacs K., Muehlendyck C., Podolski K.M., Wilke M., Heinlein W. (2022). Clinical and economic burden of surgical site infections in inpatient care in Germany: A retrospective, cross-sectional analysis from 79 hospitals. PLoS ONE.

[7] Awad S.S., Palacio C.H., Subramanian A., Byers P.A., Abraham P., Lewis D.A., Young E.J. (2009). Implementation of a methicillin-resistant Staphylococcus aureus (MRSA) prevention bundle results in decreased MRSA surgical site infections. Am. J. Surg..

[8] Weigelt J.A., Lipsky B.A., Tabak Y.P., Derby K.G., Kim M., Gupta V. (2010). Surgical site infections: Causative pathogens and associated outcomes. Am. J. Infect. Control.

[9] Frommelt L. (2018). Use of antibiotics in bones: Prophylaxis and current treatment standards. Orthopade.

[10] Chan Y.S., Ueng S.W., Wang C.J., Lee S.S., Chen C.Y., Shin C.H. (2000). Antibiotic-impregnated autogenic cancellous bone grafting is an effective and safe method for the management of small infected tibial defects: A comparison study. J. Trauma.

[11] Vardanian A.J., Chau A., Quinones-Baldrich W., Lawrence P.F. (2009). Arterial allograft allows in-line reconstruction of prosthetic graft infection with low recurrence rate and mortality. Am. Surg..

[12] Rupp M., Klute L., Baertl S., Walter N., Mannala G.K., Frank L., Pfeifer C., Alt V., Kerschbaum M. (2021). The clinical use of bone graft substitutes in orthopedic surgery in Germany-A 10-years survey from 2008 to 2018 of 1,090,167 surgical interventions. J. Biomed. Mater. Res. B Appl. Biomater..

[13] Balling H., Weckbach A. (2020). Demineralized bone matrix versus autogenous bone graft for thoracolumbar anterior single-level interbody fusion. Acta Orthopædica Belg..

[14] Di Bella C., Dozza B., Frisoni T., Cevolani L., Donati D. (2010). Injection of demineralized bone matrix with bone marrow concentrate improves healing in unicameral bone cyst. Clin. Orthop. Relat. Res..

[15] Drosos G.I., Touzopoulos P., Ververidis A., Tilkeridis K., Kazakos K. (2015). Use of demineralized bone matrix in the extremities. World J. Orthop..

[16] Kuhls R., Werner-Rustner M., Kuchler I., Soost F. (2001). Human demineralised bone matrix as a bone substitute for reconstruction of cystic defects of the lower jaw. Cell. Tissue Bank..

[17] Winkler H., Kaudela K., Stoiber A., Menschik F. (2006). Bone grafts impregnated with antibiotics as a tool for treating infected implants in orthopedic surgery—One stage revision results. Cell. Tissue Bank..

[18] Winkler H., Stoiber A., Kaudela K., Winter F., Menschik F. (2008). One stage uncemented revision of infected total hip replacement using cancellous allograft bone impregnated with antibiotics. J. Bone Jt. Surg. Br..

[19] Coraca-Huber D.C., Steixner S.J.M., Najman S., Stojanovic S., Finze R., Rimashevskiy D., Saginova D., Barbeck M., Schnettler R. (2022). Lyophilized Human Bone Allograft as an Antibiotic Carrier: An In Vitro and In Vivo Study. Antibiotics.

[20] Anagnostakos K., Kelm J. (2009). Enhancement of antibiotic elution from acrylic bone cement. J. Biomed. Mater. Res. B Appl. Biomater..

[21] McConoughey S.J., Howlin R.P., Wiseman J., Stoodley P., Calhoun J.H. (2015). Comparing PMMA and calcium sulfate as carriers for the local delivery of antibiotics to infected surgical sites. J. Biomed. Mater. Res. B Appl. Biomater..

[22] Bormann N., Schwabe P., Smith M.D., Wildemann B. (2014). Analysis of parameters influencing the release of antibiotics mixed with bone grafting material using a reliable mixing procedure. Bone.

[23] Ketonis C., Barr S., Shapiro I.M., Parvizi J., Adams C.S., Hickok N.J. (2011). Antibacterial activity of bone allografts: Comparison of a new vancomycin-tethered allograft with allograft loaded with adsorbed vancomycin. Bone.

[24] Witsø E., Persen L., Benum P., Bergh K. (2005). Cortical allograft as a vehicle for antibiotic delivery. Acta Orthop..

[25] Pruss A., Baumann B., Seibold M., Kao M., Tintelnot K., von Versen R., Radtke H., Dorner T., Pauli G., Gobel U.B. (2001). Validation of the sterilization procedure of allogeneic avital bone transplants using peracetic acid-ethanol. Biologicals.

[26] Diefenbeck M., Mückley T., Hofmann G.O. (2006). Prophylaxis and treatment of implant-related infections by local application of antibiotics. Injury.

[27] Winkler H., Haiden P. (2017). Allograft Bone as Antibiotic Carrier. J. Bone Jt. Infect..

[28] Sabbagh F., Kim B.S. (2022). Microneedles for transdermal drug delivery using clay-based composites. Expert Opin. Drug. Deliv..

[29] Van Vugt T.A.G., Arts J.J., Geurts J.A.P. (2019). Antibiotic-Loaded Polymethylmethacrylate Beads and Spacers in Treatment of Orthopedic Infections and the Role of Biofilm Formation. Front. Microbiol..

[30] Buttaro M.A., Pusso R., Piccaluga F. (2005). Vancomycin-supplemented impacted bone allografts in infected hip arthroplasty. Two-stage revision results. J. Bone Jt. Surg. Br..

[31] Witsø E., Persen L., Løseth K., Bergh K. (1999). Adsorption and release of antibiotics from morselized cancellous bone. In vitro studies of 8 antibiotics. Acta Orthop. Scand..

[32] Witso E., Persen L., Loseth K., Benum P., Bergh K. (2000). Cancellous bone as an antibiotic carrier. Acta Orthop. Scand..

[33] Gruskin E., Doll B.A., Futrell F.W., Schmitz J.P., Hollinger J.O. (2012). Demineralized bone matrix in bone repair: History and use. Adv. Drug Deliv. Rev..

[34] Hou H., Li B., Zhang Z., Xue C., Yu G., Wang J., Bao Y., Bu L., Sun J., Peng Z. (2012). Moisture absorption and retention properties, and activity in alleviating skin photodamage of collagen polypeptide from marine fish skin. Food Chem..

[35] Thitiset T., Damrongsakkul S., Bunaprasert T., Leeanansaksiri W., Honsawek S. (2013). Development of collagen/demineralized bone powder scaffolds and periosteum-derived cells for bone tissue engineering application. Int. J. Mol. Sci..

[36] Sohling N., Leiblein M., Schaible A., Janko M., Schwable J., Seidl C., Brune J.C., Nau C., Marzi I., Henrich D. (2020). First Human Leucocyte Antigen (HLA) Response and Safety Evaluation of Fibrous Demineralized Bone Matrix in a Critical Size Femoral Defect Model of the Sprague-Dawley Rat. Materials.

[37] Huang X., Brazel C.S. (2001). On the importance and mechanisms of burst release in matrix-controlled drug delivery systems. J. Control. Release.

[38] Borcherding K., Marx D., Gatjen L., Bormann N., Wildemann B., Specht U., Salz D., Thiel K., Grunwald I. (2019). Burst Release of Antibiotics Combined with Long-Term Release of Silver Targeting Implant-Associated Infections: Design, Characterization and in vitro Evaluation of Novel Implant Hybrid Surface. Materials.

[39] Sorensen T.S., Sorensen A.I. (1993). Bactericidal activity of gentamicin against S. aureus. In vitro study questions value of prolonged high concentrations. Acta Orthop. Scand..

[40] Ueng S.W., Hsieh P.H., Shih H.N., Chan Y.S., Lee M.S., Chang Y. (2012). Antibacterial activity of joint fluid in cemented total-knee arthroplasty: An in vivo comparative study of polymethylmethacrylate with and without antibiotic loading. Antimicrob. Agents Chemother..

[41] Eidem T.M., Coughlan A., Towler M.R., Dunman P.M., Wren A.W. (2014). Drug-eluting cements for hard tissue repair: A comparative study using vancomycin and RNPA1000 to inhibit growth of Staphylococcus aureus. J. Biomater. Appl..

[42] Von Stechow D., Rauschmann M. (2009). Effectiveness of combination use of antibiotic-loaded PerOssal^®^ with spinal surgery in patients with spondylodiscitis. Eur. Surg. Res..

[43] Beuttel E., Bormann N., Pobloth A.M., Duda G.N., Wildemann B. (2019). Impact of Gentamicin-Loaded Bone Graft on Defect Healing in a Sheep Model. Materials.

