# Supplementary material for: Analysis of the Ability of Different Allografts to Act as Carrier Grafts for Local Drug Delivery
Source: J Funct Biomater. 2023 Jun 1;14(6):305. doi: 10.3390/jfb14060305 (PMC10299409; doi:10.3390/jfb14060305)
Supplement: Supplementary file 1 [file jfb-14-00305-s001.zip › jfb-2416030 supplementary material.pdf]

*Article*

# Analysis of the Ability of Different Allografts to Act as Carrier Grafts for Local Drug Delivery

**Nicole Bormann [1], Aysha Schmock [1], Anja Hanke [2], Volker Eras [2], Norus Ahmed [2,*], Maya S. Kissner [1], Britt Wildemann [1,3] and Jan C. Brune [2]**

[1] Julius Wolff Institut und BIH-Center für Regenerative Therapien und Center, Charité-Universitätsmedizin Berlin, Corporate Member of Freie Universität Berlin, Humboldt-Universität zu Berlin und Berlin Institute of Health, 13353, Berlin, Germany; nicole.bormann@bih-charite.de (N.B.); aysha.bonell@bih-charite.de (A.S.); maya.kissner@web.de (M.S.K.); britt.wildemann@med.uni-jena.de (B.W.)

[2] German Institute for Cell and Tissue Replacement (DIZG, gemeinnützige GmbH), 12555 Berlin, Germany; a_hanke@dizg.de (A.H.); v_eras@dizg.de (V.E.); j_brune@dizg.de (J.C.B.)

[3] Experimental Trauma Surgery, Department of Trauma, Hand and Reconstructive Surgery, Jena University Hospital, Friedrich Schiller University, 07747 Jena, Germany

* Correspondence: n_ahmed@dizg.de

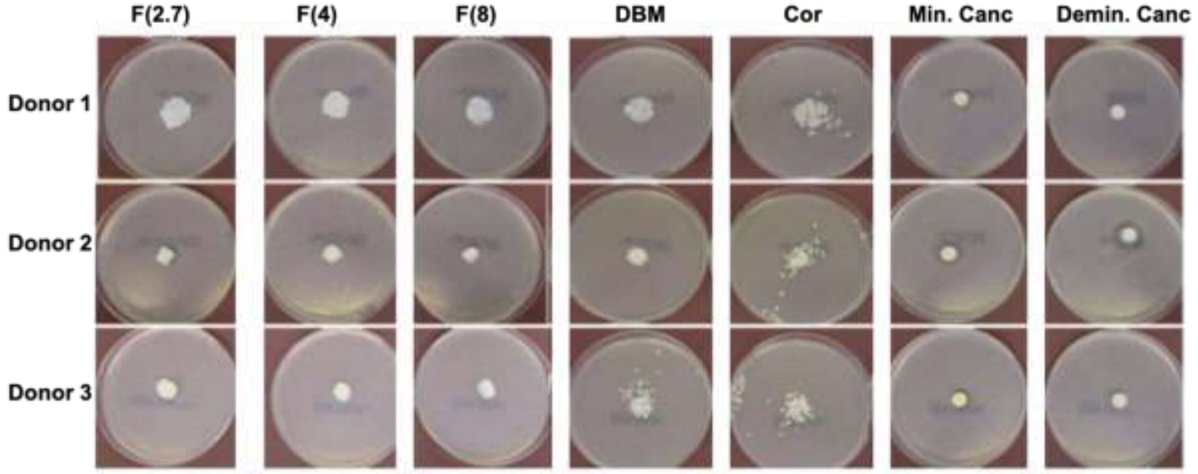


**Supplementary Figure S1.** Zone of inhibition (ZOI) test after 30 minutes of incubation: Gentamicin elution 3 from the respective grafts after 21 days of elution.

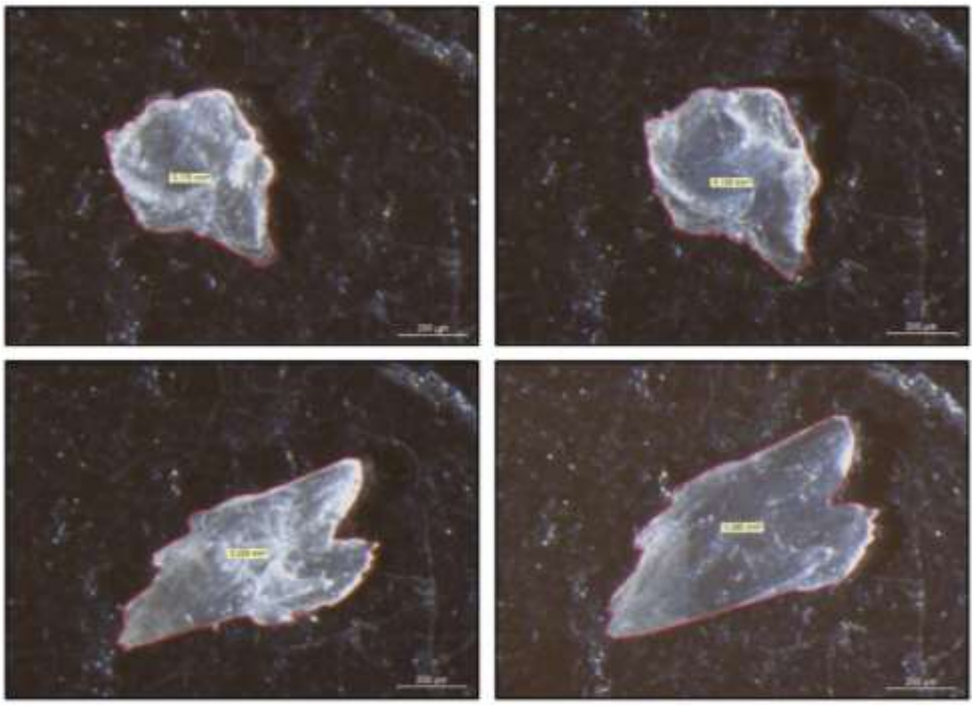

**Supplementary Figure S2:** Microscopy images of DBM particles before (A and C, left) and after (B and D, 23 right) rehydration with water (6.3 x magnification, scale bar = 200 µm).

**Supplementary Table S1.** Grafts *used for the absorption capacity testing with PBS*

| Group | Type | Dry weight (mg, mean ± SD) |
|---|---|---|
| Cancellous bone (high density) | Mineralized | 216 ± 29 |
| Cancellous bone (medium density) | Mineralized | 126 ± 3 |
| Cancellous bone (low density) | Mineralized | 61 ± 6 |
| Cancellous bone | Demineralized | 55 ± 22 |
| Cortical granules (1-2 mm) | Mineralized | 282 ± 1 |
| DBM granules (1-2 mm) | Demineralized | 106 ± 1 |
| Fibrous graft (2.7 ml/g) | Demineralized | 141 ± 1 |
| Fibrous graft (4 ml/g) | Demineralized | 102 ± 0 |

**Supplementary Table S2.** Grafts used *for the gentamicin elution testing*

| Group | Type | Dry weight (mg, mean ± SD) | Antibiotic addition (µL) |
|---|---|---|---|
| Cancellous bone | Mineralized | 171.4 ± 22.4 | 2000 |
| Cancellous bone | Demineralized | 60.7 ± 6.8 | 2000 |
| Cortical granules (1-2 mm) | Mineralized | 344.4 ± 2.9 | 2000 |
| DBM granules (1-2 mm) | Demineralized | 104.3 ± 1.2 | 2000 |
| Fibrous graft (2.7 ml/g) | Demineralized | 91.9 ± 2.4 | 243 |
| Fibrous graft (4 ml/g) | Demineralized | 76.5 ± 1.1 | 300 |
| Fibrous graft (8 ml/g) | Demineralized | 46.2 ± 1.8 | 360 |

**Supplementary Table S3.** Statistical significance of total fluid absorbed in the matrix (lower part in red) and interstitial spaces (upper part in blue). Given are the exact p-values. Fibrous grafts absorbed significantly more fluid than any other group.

| | Fibrous graft (2.7 ml/g) | Fibrous graft (4 ml/g) | DBM granules (1-2 mm) | Cortical granules (1-2 mm) | Min. Cancellous bone(low density) | Min. Cancellous bone (medium density) | Min. Cancellous bone (high density) | Demin. Cancellous bone |
|---|---|---|---|---|---|---|---|---|
| Fibrous graft (2.7 ml/g) | | <0.0001 | <0.0001 | <0.0001 | <0.0001 | <0.0001 | <0.0001 | <0.0001 |
| Fibrous graft (4 ml/g) | <0.0001 | | 0.8114 | 0.9985 | <0.0001 | <0.0001 | 0.0071 | <0.0001 |
| DBM granules (1-2 mm) | <0.0001 | <0.0001 | | 0.987 | <0.0001 | <0.0001 | 0.1782 | <0.0001 |
| Cortical granules (1-2 mm) | <0.0001 | <0.0001 | <0.0001 | | <0.0001 | <0.0001 | 0.0305 | <0.0001 |
| Min. Cancellous bone (low density) | <0.0001 | <0.0001 | <0.0001 | 0.0003 | | 0.9997 | <0.0001 | 0.814 |
| Min. Cancellous bone (medium density) | <0.0001 | <0.0001 | <0.0001 | 0.4639 | 0.1176 | | 0.0007 | 0.6272 |
| Min. Cancellous bone (high density) | <0.0001 | <0.0001 | <0.0001 | 0.9998 | 0.0005 | 0.3338 | | <0.0001 |
| Demin. Cancellous bone | <0.0001 | <0.0001 | <0.0001 | 0.0004 | <0.0001 | <0.0001 | 0.0043 | |

**Supplementary Table S4.** Statistical significance of gentamicin elution by different bony grafts over a 21-day period. Given are the exact p-values. No significant differences were observed after day 7. ns: not significant ($p > 0.05$); F(2.7): Fibrous graft 2.7 ml/g, F(4): Fibrous graft 4 ml/g; F(8): Fibrous graft 8 ml/g; Cor: cortical granules; Min. Canc: cancellous bone; DBM: demineralized bone matrix; Demin. Canc: demineralized cancellous bone.

| Parameters | 1h | 4h | 1d | 2d | 3d | 4d | 7d |
|---|---|---|---|---|---|---|---|
| F(2.7) vs. F(4) | ns | ns | ns | ns | ns | ns | ns |
| F(2.7) vs. F(8) | 0.0166 | ns | ns | ns | 0.0207 | 0.0069 | 0.0365 |
| F(2.7) vs. DBM | ns | 0.0041 | ns | ns | 0.0209 | ns | ns |
| F(2.7) vs. Cor | ns | 0.0006 | ns | ns | 0.0268 | 0.0009 | 0.0419 |
| F(2.7) vs. Min. Canc | ns | ns | ns | ns | 0.0465 | ns | ns |
| F(2.7) vs. Demin. Canc | ns | 0.0046 | ns | ns | 0.0142 | 0.0003 | ns |
| F(4) vs. F(8) | 0.0297 | ns | 0.0321 | 0.03 | 0.0156 | 0.042 | 0.0229 |
| F(4) vs. DBM | ns | 0.0021 | 0.0322 | 0.0339 | ns | ns | ns |
| F(4) vs. Cor | ns | 0.0007 | 0.0372 | 0.0398 | 0.0248 | 0.0353 | 0.0089 |
| F(4) vs. Min. Canc | ns | 0.0319 | 0.0488 | ns | ns | ns | ns |
| F(4) vs. Demin. Canc | ns | 0.0027 | 0.0338 | 0.0216 | 0.0244 | ns | 0.0143 |
| F(8) vs. DBM | ns | ns | ns | ns | ns | ns | ns |
| F(8) vs. Cor | 0.0193 | ns | ns | ns | ns | ns | ns |
| F(8) vs. Min. Canc | 0.0343 | ns | ns | ns | 0.0292 | ns | ns |
| F(8) vs. Demin. Canc | ns | ns | ns | ns | ns | ns | ns |
| DBM vs. Cor | ns | ns | ns | ns | ns | ns | ns |
| DBM vs. Min. Canc | ns | ns | ns | ns | ns | ns | ns |
| DBM vs. Demin. Canc | ns | ns | ns | ns | ns | ns | ns |
| Cor vs. Min. Canc | ns | ns | ns | 0.0147 | 0.0075 | ns | ns |
| Cor vs. Demin. Canc | ns | ns | ns | ns | ns | ns | ns |
| Can vs. Demin. Canc | ns | ns | ns | ns | ns | ns | ns |

**Supplementary Table S5.** Statistical significance of ZOI testing with different bony grafts over a 21-day period. Given are the exact p-values. No significant differences were observed after day 3. ns: not significant ($p > 0.05$); F(2.7): Fibrous graft 2.7 ml/g, F(4): Fibrous graft 4 ml/g; F(8): Fibrous graft 8 ml/g; Cor: cortical granules; Min. Canc: cancellous bone; DBM: demineralized bone matrix; Demin. Canc: demineralized cancellous bone.

| Parameters | 1h | 4h | 1d | 2d | 3d |
|---|---|---|---|---|---|

| | | | | | |
|---|---|---|---|---|---|
| F(2.7) vs. F(4) | ns | ns | ns | ns | ns |
| F(2.7) vs. F(8) | ns | ns | 0.0379 | ns | 0.0137 |
| F(2.7) vs. DBM | ns | 0.0033 | 0.0028 | ns | ns |
| F(2.7) vs. Cor | ns | ns | 0.0231 | 0.027 | 0.0137 |
| F(2.7) vs. Min. Canc | ns | ns | 0.0468 | ns | ns |
| F(2.7) vs. Demin. Canc | ns | ns | ns | ns | 0.0137 |
| F(4) vs. F(8) | ns | ns | 0.0489 | ns | 0.0142 |
| F(4) vs. DBM | ns | 0.0021 | 0.0063 | ns | ns |
| F(4) vs. Cor | ns | ns | 0.0244 | ns | ns |
| F(4) vs. Min. Canc | ns | ns | ns | ns | ns |
| F(4) vs. Demin. Canc | ns | ns | ns | ns | 0.0142 |
| F(8) vs. DBM | ns | ns | ns | ns | ns |
| F(8) vs. Cor | ns | ns | ns | ns | 0 |
| F(8) vs. Min. Canc | ns | ns | ns | ns | 0.0275 |
| F(8) vs. Demin. Canc | ns | ns | ns | ns | ns |
| DBM vs. Cor | ns | ns | ns | ns | ns |
| DBM vs. Min. Canc | ns | 0.0487 | ns | ns | ns |
| DBM vs. Demin. Canc | ns | 0.0387 | ns | ns | ns |
| Cor vs. Min. Canc | ns | ns | ns | ns | 0.0275 |
| Cor vs. Demin. Canc | ns | ns | ns | ns | ns |
| Can vs. Demin. Canc | ns | ns | ns | ns | 0.0275 |

**Supplementary Table S6.** Statistical significance of gentamicin elution of Fibrous graft 2.7 ml/g with different incubation times over a 21-day period. Given are the exact p-values. No significant differences were observed after day 1. ns: not significant (p > 0.05).

| **Parameters** | **1h** | **4h** | **1d** | **2d** |
|---|---|---|---|---|
| 5 min vs. 10 min | <0.0001 | 0.0063 | ns | ns |
| 5 min vs. 20 min | ns | ns | ns | ns |
| 5 min vs. 30 min | <0.0001 | 0.036 | 0.0003 | ns |
| 10 min vs. 20 min | <0.0001 | ns | ns | ns |
| 10 min vs. 30 min | ns | ns | 0.0014 | ns |
| 20 min vs. 30 min | <0.0001 | ns | 0.0058 | ns |
